# Integrative methods reveal multiple drivers of diversification in rice paddy snakes

**DOI:** 10.1038/s41598-024-54744-z

**Published:** 2024-03-12

**Authors:** Justin M. Bernstein, Harold K. Voris, Bryan L. Stuart, Daryl R. Karns, Jimmy A. McGuire, Djoko T. Iskandar, Awal Riyanto, Camilo A. Calderón-Acevedo, Rafe M. Brown, Marcelo Gehara, J. Angel Soto-Centeno, Sara Ruane

**Affiliations:** 1 Center for Genomics, University of Kansas, Dyche Hall, 1345 Jayhawk Blvd, Lawrence, KS 66045 USA; 2 Life Sciences Section, Negaunee Integrative Research Center, Field Museum, 1400 S. Lake Shore Drive, Chicago, IL 60605 USA; 3 Section of Research and Collections, North Carolina Museum of Natural Sciences, Raleigh, NC 27601 USA; 4 Biology Department, Hanover College, Hanover, IN 47243 USA; 5 Museum of Vertebrate Zoology and Department of Integrative Biology, University of California, Berkeley, CA 94720 USA; 6 School of Life Sciences and Technology, Institut Teknologi Bandung, Bandung, Indonesia; 7 Museum Zoologicum Bogoriense, Research Center for Biology, National Research and Innovation Agency of Indonesia (BRIN), Cibinong, 16911 Indonesia; 8 State University of New York: College of Environmental Science and Forestry, Syracuse, NY 13210 USA; 9 Department of Ecology and Evolutionary Biology and Biodiversity Institute, University of Kansas, Lawrence, KS 66045 USA; 10 Department of Earth and Environmental Science, Rutgers University-Newark, Newark, NJ 07102 USA; 11 Department of Mammalogy, American Museum of Natural History, New York, NY 10024 USA

**Keywords:** Phylogenetics, Speciation, Population genetics

## Abstract

Divergence dating analyses in systematics provide a framework to develop and test biogeographic hypotheses regarding speciation. However, as molecular datasets grow from multilocus to genomic, sample sizes decrease due to computational burdens, and the testing of fine-scale biogeographic hypotheses becomes difficult. In this study, we use coalescent demographic models to investigate the diversification of poorly known rice paddy snakes from Southeast Asia (Homalopsidae: *Hypsiscopus*), which have conflicting dates of origin based on previous studies. We use coalescent modeling to test the hypothesis that *Hypsiscopus* diversified 2.5 mya during the Khorat Plateau uplift in Thailand. Additionally, we use ecological niche analyses to identify potential differences in the niche space of the two most widely distributed species in the past and present. Our results suggest *Hypsiscopus* diversified ~ 2.4 mya, supporting that the Khorat Plateau may have initiated the diversification of rice paddy snakes. We also find significant niche differentiation and shifts between species of *Hypsiscopus*, indicating that environmental differences may have sustained differentiation of this genus after the Khorat Plateau uplift. Our study expands on the diversification history of snakes in Southeast Asia, and highlights how results from smaller multilocus datasets can be useful in developing and testing biogeographic hypotheses alongside genomic datasets.

## Introduction

Widely distributed taxa can serve as excellent model systems to test biogeographic hypotheses, especially in regions with complex geologic histories and a mosaic of geological features. One group that includes several wide-ranging taxa, in one of the most complex regions are the mud snakes; Old World mud snakes of the family Homalopsidae consist of 57 species in 26 genera distributed throughout South and Southeast Asia, Australia, and New Guinea^1^. Homalopsids are found across aquatic environments with muddy substrates and varying salinities (e.g., rice paddies, tidal flats, mangroves, swamp forests, freshwater lakes and streams)^2^. These mud snakes exhibit a variety of feeding behaviors^3^, diets^4^ and morphological adaptations such as rostral mechanoreceptors^5^ and salt glands^6^. Recently, phylogenetic studies with multilocus datasets have expanded on the evolutionary relationships and divergence times of Homalopsidae^7^. A few genera of mud snakes are extremely abundant in aquatic systems of Southeast Asia and have some of the most widespread distributions of any terrestrial vertebrate^1,2^. This makes them ideal for investigating how Southeast Asia’s complex geologic history^8,9^ has generated patterns of diversity observed today.

**Figure 1:**
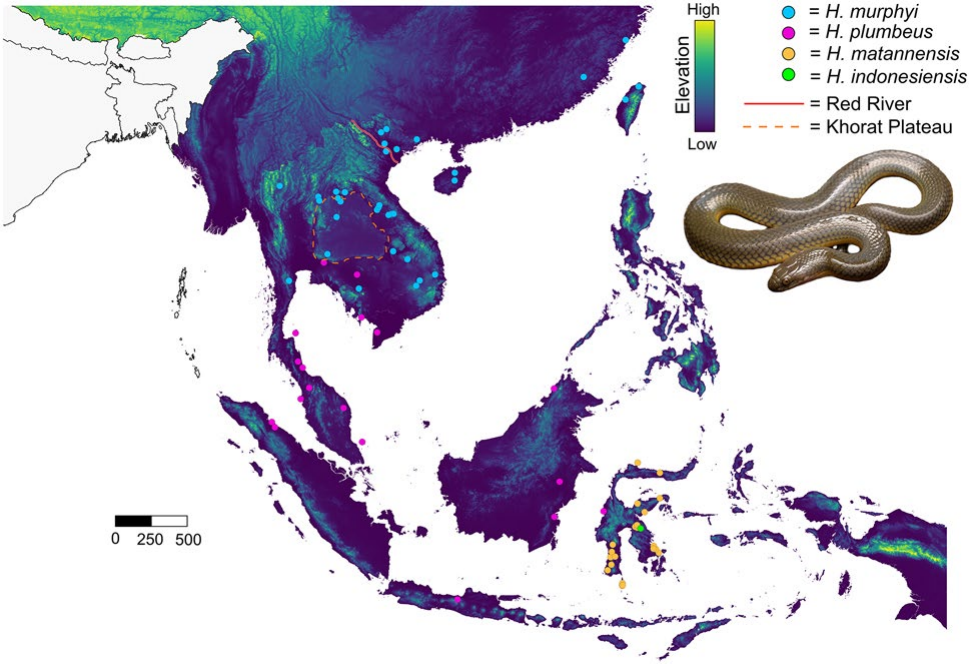
Sampling map of *Hypsiscopus* in this study. Map is colored by elevation (0–4509 m). The Red River and Khorat Plateau boundary are shown by the solid red line and dashed orange line, respectively. Photograph of *H. murphyi* by BLS.

Rice paddy snakes of the genus *Hypsiscopus* consist of four species. The newly described *Hypsiscopus murphyi* is distributed around and north of Thailand’s Khorat Plateau, and *H. plumbeus* is found around and south of the Khorat Plateau, and on the Indonesian islands as far east as Sulawesi^7^. *Hypsiscopus matannensis* and *H. indonesiensis* are endemic to Sulawesi, the eastern limit of the distribution of the genus (Fig. 1). Phylogenetic investigations of homalopsids using mitochondrially-driven multilocus data have placed the *Hypsiscopus* origin at ~ 2.5 million years ago (mya)^7^. Thus, it is hypothesized that the tectonic uplift event that led to the Khorat Plateau at ~ 2.5 mya^9,10^ may have been responsible for the diversification of rice paddy snakes^7^. However, date estimates of recent systematic works of Homalopsidae using genomic data pre-date the formation of the plateau with an estimated divergence of ~ 4 mya. These two studies^7,11^, each yielding conflicting divergence dates around the Khorat Plateau hypothesis, use different depths of DNA sequencing and different divergence date estimation techniques.

Estimates of clade divergence timing on molecular phylogenies are standard in systematic studies^12^, especially parameter-heavy approaches that use model-based likelihood methods^13,14^ which have helped in formulating and testing biogeographic hypotheses. Modern phylogenetics now frequently use genomic datasets that include thousands of loci, but this comes at the cost of major increases in computational burden and analysis run times. Potentially alleviating some of these costs, summary coalescent methods aim to reconstruct phylogenies rapidly and estimate divergence dates (e.g., ASTRAL^15^; treePL^16^). Because of a higher number of informative sites and the potential to characterize evolutionary heterogeneity across independent genes, a greater number of loci is presumed to increase accuracy in divergence time estimates. Compared to single-gene or small multilocus datasets dated using computationally rigorous Bayesian methods like BEAST^17^, phylogenomic datasets are assumed to provide a better representation of species divergence and, as such, may allow for finer scale temporal discrimination of alternate hypotheses in biogeography^18^. However, the methods used to rapidly obtain divergence dates from genomic datasets may not be useful in a hypothesis-testing framework for particular geological events. This is due to genomic studies typically having lower sample sizes due to costs, thus possibly missing important population-level data, or because of the computation times associated with analysis of large datasets.

Methods based on coalescent simulations of demographic models can be more intuitively customized to the study taxon to test specific evolutionary scenarios^19^. Coalescent models can incorporate multilocus or genomic datasets and can be fine-tuned to test phylogeographic hypotheses with large sample sizes. Obtaining accurate divergence dates is critical for downstream studies that aim to identify the causal factors of population splits and speciation, especially at potential biogeographic barriers^20^. Herein, we examine whether rice paddy snake diversification coincides with the rise of the Khorat Plateau and test for differences in each species’ environmental niche. The expansive range of the non-sister species *H. murphyi* and *H. plumbeus* (1.7 million km^2^ for *H. murphyi* and ~ 4 million km^2^ for *H. plumbeus*) means that they span a heterogeneous landscape of environmental, habitat, and topographic features. Phylogeographic structure and the maintenance of species limits driven by environmental heterogeneity has been documented in widely distributed taxa^21–23^, including areas that contain well-known biogeographic barriers^24,25^. Given the wide distribution of rice paddy snakes, we examine if quantifiable differences in the environmental niches reflect phylogeographic structure in these snakes^26^.

In this study, we used newly obtained sequence data for *Hypsiscopus* to investigate the diversification of rice paddy snakes through Southeast Asia. We obtained 303 new DNA sequences of 6 nuclear and 2 mitochondrial genes, and combined these with previously published data to generate a dataset consisting of 653 sequences across 8 genes for 151 individuals. Using coalescent demographic models with priors that encompass known *Hypsiscopus* evolutionary history, we tested the hypothesis that the crown group of *Hypsiscopus* diversified ~ 2.5 mya when the tectonic uplift of the Khorat Plateau took place^9,10^. *Hypsiscopus* is one of few taxa that have parapatric distributions around the plateau and are abundant enough to obtain a dense sampling scheme for evolutionary studies, making them an ideal system to test our diversification hypothesis. We aim to identify modes of speciation in Southeast Asia, which are often referred to in regards to the prevailing paradigm in this region: Pleistocene sea-level fluctuations. We performed quantitative morphological, molecular phylogenetic, and ecological analyses to investigate homalopsid phenotypic and genetic diversity, and predict that environmental data will yield quantifiable niche differences between the two widespread species: *H. murphyi* and *H. plumbeus*.

## Methods

### Taxon sampling, DNA extraction, and DNA sequencing

We sampled 42 *H. plumbeus*, 120 *H. murphyi*, and 21 *Hypsiscopus matannensis* throughout their geographic ranges, totaling 183 specimens (Fig. 1). This includes 24 individuals of *H. murphyi* near and east of the Red River in Vietnam and 15 individuals of *H. matannensis* from Sulawesi, which are populations that were under-sampled in previous homalopsid studies^7,11^. Although snakes were collected from previous expeditions (i.e., not for this study) and were available through natural history loans, we nonetheless report the appropriate permits for the original field collections. Snakes collected in the field by authors were humanely handled following Herpetological Animal Care and Use Committee (HACC) of the American Society of Ichthyologists and Herpetologists (2004; https://www.asih.org/resources) and in compliance with Institutional Animal Care and Use Committee (IACUC) protocols FMNH 06-4 to HKV and NCSM 2011-01 to BLS. Research and export permits to JAM were issued by LIPI and RISTEK-DIKTI, and AUP #R279 issued by the UC Berkeley IACUC. All experimental protocols were approved by the Field Museum of Natural History, North Carolina Museum of Natural Science, and the University of California, Berkeley. All experiments were performed in accordance with relevant guidelines and regulations, and all protocols are in accordance with the ARRIVE guidelines. Snakes were euthanized by cardiac injection of tricaine methanesulfonate (MS222) or Nembutal (pentobarbital sodium) and fixed in 10% buffered formalin after preserving liver or muscle tissue samples in ≥ 95% ethanol, RNAlater, or DMSO-salt saturated storage buffer. Specimens were later transferred to 70% ethanol and deposited at the Field Museum of Natural History, Museum of Vertebrate Zoology, North Carolina Museum of Natural Sciences, National University of Laos, and Texas Natural History Collections (TNHC). Additional tissues were obtained from these institutions as well as the natural history collections at the University of Kansas Natural History Museum and Biodiversity Institute, Florida Museum of Natural History, American Museum of Natural History, La Sierra University Herpetological Collection, Sabah State Museum, Royal Ontario Museum, Smithsonian National Museum of Natural History, Yale Peabody Museum, and Museum of Comparative Zoology.

Whole genomic DNA was extracted from liver and muscle tissue using Qiagen DNeasy Blood & Tissue Kits, following the standard tissue protocol. We amplified two mitochondrial genes and six nuclear genes. For mitochondrial genes, a continuous 849–882 bp fragment composed of the tRNA-Lys gene, the complete ATPase subunit 6 and subunit 8 genes, and part of the cytochrome oxidase c subunit III gene (hereafter as “ATPase”) was amplified. We also amplified a fragment spanning a continuous region corresponding to the tRNA-Glu gene, the complete cytochrome b gene, and a segment of the tRNA-Thr gene (collectively referred to as “cyt-b”). For the nuclear genes, we amplified a 553 bp fragment of the prolactin receptor (PRLR), a ~ 1300 bp fragment of the WAP, Follistatin/Kazal, Immunoglobulin, Kunitz and Netrin Domain Containing 2 (WFIKKN2) gene, an 855 bp fragment of the vacuolar protein sorting-associated protein 13B (VPS13B) gene, a ~ 627 bp fragment of ATP/GTP binding protein-like 5 (AGBL5), a ~ 1949 bp fragment of zinc finger protein basonuclin-1 (BNC1), and a ~ 1100 bp fragment of recombination activating gene 2 (RAG2). We amplified cyt-b, ATPase, and PRLR using the polymerase chain reaction (PCR) protocols of Bernstein et al.^27^. We followed nested PCR protocols^28,29^ to amplify WFIKKN, VPS13B, AGBL5, BNC1, and RAG2. PCR products were visualized on a 1.5% agarose gel, and amplicons were cleaned with ExoSAP-IT (Applied BioSystems). Cleaned PCR products were sequenced on a 3730 DNA Analyzer (Applied Biosystems) using Big-Dye Terminator version 3 chemistry (Applied Biosystems) and amplifying and sequencing primers. All amplification and sequencing primers were developed from previous studies^28–31^ and can be found in Supplementary Table S1. We supplemented our sequencing efforts with additional sequences of cyt-b, ATPase, PRLR, WFIKKN2, and VPS13B from previous studies on *Hypsiscopus*^7,27^ from NCBI’s Genbank. Genbank Accession IDs for all samples in this study can be found in Supplementary Table S1 and primers for amplification and sequencing in Supplementary Table S2.

### Phylogenetic analyses

We aligned and concatenated genes using the Geneious alignment algorithm in Geneious R11.1.5 under default parameters. Because the interspecific relationships of *Hypsiscopus* have already been supported in prior studies using coalescent methods with multilocus and genomic datasets^7,11,27^, we concatenated our gene alignments to assess the monophyly and population structure of newly sequenced *Hypsiscopus* samples in this study. We manually edited alignments by eye and checked for ambiguous base calls for sequencing errors. To better understand the distribution of alleles in populations, we called heterozygotes using the PHASE function in DnaSP6^32^ using default parameters. Maximum likelihood gene trees and a concatenated phylogeny were reconstructed using IQ-TREE v1.6.1^28^ using 1000 ultrafast bootstrap (UFB)^29,30^ iterations for statistical support. We also implemented 1000 bootstrap replicates of the SH-like approximate likelihood-ratio test (SH-aLRT) for additional support values^33,34^. We consider relationships that have UFB ≥ 95 and SH-aLRT values ≥ 80 to be strongly supported^33^. To determine and implement the best DNA substitution model for each run, we used the ModelFinder^35^ function in IQ-TREE. We ran the concatenated phylogenetic reconstruction using the same methods and parameters as above, but also used PartitionFinder2^36^ to determine the best partitioning scheme for our data, partitioning by mitochondrial genes and nuclear genes separately, and the latter by codon position. Phylogenetic reconstruction was performed for unphased and phased alignments. Following the phylogeny from Bernstein et al. (2021)^7^, we used sequences of *Homalopsis buccata*, *Homalopsis nigroventralis*, *Myrrophis chinensis*, and *Enhydris chanardi* from other studies^7,30,37^ to designate as outgroups. All trees were visualized using FigTree v1.3.1^38^.

### Morphological quantitative statistics

Previous investigations^27^ of the morphological diversity of *Hypsiscopus* used methods that required no a priori groupings (e.g., principal coordinate analysis; PCA). While we also construct a PCA in this study to investigate morphological diversity, we also employed machine learning linear discriminant analysis (LDA) to examine morphological variation using a priori groupings from Bernstein et al.^27^ at the species and population-levels of *H. plumbeus* and *H. murphyi*. The purpose of this is to use supervised and unsupervised machine learning classification models to test phenotypic boundaries and better understand inter-group structure in morphospace. We kept *H. plumbeus* as a single taxonomic entity in our analysis, while splitting *H. murphyi* into two clusters that are phenotypically distinct based on color pattern: one representing a population east of the Red River in Indochina, and the other representing a population west of this large biogeographic barrier^39,40^. All individuals were assigned to the species or population group based on raw morphological characteristics^27^ (Supplementary Table S1) and molecular phylogenetics (see *Results*).

We analyzed all morphological data in R v.4.0.2^41^ using the *dplyr*^42^, *vegan*^43^, *labdsv*^44^, *ggbiplot*^45^, *mice*^46^, *car*^47^, *MASS*^48^, *caret*^49,50^, and base *stats* packages. The LDA creates a confusion matrix to assess the prediction accuracy of a priori groupings. We converted categorical data (e.g., color pattern) to numerical values representing discrete states, and then log transformed the dataset. Thus, our morphological dataset consisted of 31 variables comprised of meristic (scale counts), categorical, and continuous (e.g., size) data. We considered individuals < 300 mm total length as juveniles^51^, and excluded them from the LDA. Overall, we examined 44 *H. murphyi* west of the Red River, 21 *H. murphyi* from and east of the Red River, and 21 *H. plumbeus* in this LDA analysis. Phenotypic data for the biogeographically isolated *H*. *matannensis* was not included for simplicity of interpretation, as it only co-occurs with *H. plumbeus,* and *H. indonesiensis* is endemic to Sulawesi. Code and input data for the LDA are available in Supplementary Data D1–D3.

### Coalescent demographic modeling

To determine divergence dates and demographic histories of *Hypsiscopus*, we used the R package *PipeMaster*^52,53^, a coalescent modeling wrapper package in R that uses *ms*^54^, *msABC*^55,56^ and *caret*^49,50^ packages. The program *ms* simulates coalescent trees under the Wright-Fisher model, and places segregating sites on these trees under the infinite sites model. We retained the same structure of the empirical data, number of samples, and base pair length to simulate nucleotide sequences under six alternative demographic scenarios: (1) Isolation with no demographic change or migration (Is), (2) isolation with migration and no demographic change (Im), (3) isolation with demographic change and no migration (Isd), (4) isolation with demographic change and migration (Imd), (5) a founder event with no migration (Ifd), and 6) a founder event with migration (Ifmd). Demographic change for these models is defined by a change in effective population sizes (*N*_*e*_). The last two models test for support of evolutionary scenarios that indicate dispersal from mainland Southeast Asia into the Greater Sunda Islands, and then into Sulawesi (i.e., stepping stone dispersal with bottleneck events). We used the nucleotide sequences for cyt-b and all six phased nuclear genes. Parsimony informative sites (pis) were calculated using the pis function in *phyloch*^57^ R package. The number of samples in each alignment and pis are given parenthetically for the following genes: cyt-b (118 samples | 113 pis), BNC1 (53 | 35), PRLR (96 | 22), WFIKKN (62 | 30), RAG2 (48 | 37), VPS13B (80 | 35), and AGBL5 (65 | 23). Demographic models were set up with the species-level topology: (*H. murphyi*, (*H. plumbeus*, *H. matannensis*)). Using cytochrome-b, we generated priors on *N*_*e*_ using Bayesian Skyline Plots (BSP) in BEAST2 under default parameters and following the vignette under BEAST documentation^17^. Estimating ancestral population sizes can be difficult to assess accurately and efficiently, so we used our BSP results as a rough indicator of differences in population size for our models (See Supplementary Fig. S1). We set a range for *N*_*e*_, with minimum and maximum *N*_*e*_ as 100,000 and 1,500,000 for *H. matannensis* and *H. plumbeus*, and 500,000 and 2,000,000 for *H. murphyi*. Divergence times parameters were given a range of 1 million to 6 million years to fully encompass the dates obtained from prior studies (~ 2.5–4.0 mya^7,11^). We set time and population size conditions on the models so the divergence time of *H. matannensis* and *H. plumbeus* was younger than the initial diversification of *H. murphyi* from the other two species. Priors for population change in the models that allow for demographic change were estimated using the BSP. Because our BSP results suggested changes in the last ~ 500,000 years (coinciding with the last 500,000 years of climate change since the Marine Oxygen Isotope Stage 12 [MIS-12]), we allowed for change in *N*_*e*_ from 10,000 to 500,000 years before present. Mutation rates were set to 1 × 10^–08^ for mitochondrial data and a range of 1.5 × 10^–09^–5 × 10^–10^ for nuclear loci. Each model was run with 50,000 simulations, and Approximate Bayesian Computation (ABC) with a tolerance value of 0.001 was used to identify the model with the greatest support; cross validation analysis was also used to verify the accuracy of model classification in the simulated data. To estimate the summary statistics of the model with the greatest support, we ran the data an additional 100,000 times, for a total of 150,000 simulations. We plotted PCA of the simulations against the empirical data to ensure model fit and that observed data falls within the simulated data (Supplementary Fig. S2). The resulting divergence dates were compared to those of previous studies obtained from cyt-b^7^ using BEAST2 and a genomic dataset^11^ using a combination of polynomial time species tree reconstruction in ASTRAL-III and a penalized likelihood approach (treePL). Code, phased alignment input files, and models for coalescent demographic models can be found in Supplementary Data D4–D18.

### Ecological niche and niche shift analysis

To determine the impact of the environment on present day distributions of *Hypsiscopus*, we constructed ecological niche models using Maxent v. 3.4.3^58,59^ following a custom R code (ENMpipe)^60^ that uses the R packages *rJava*^61^, *ENMeval*^62^, *ecospat*^63^, *dismo*^64^, *sf*^65^, *tidyverse*^66^, *dplyr*, *rgdal*^67^, *maptools*^68^, *maps*^69^, *raster*^70^, *rasterVis*^71^, *RColorBrewer*^72^, *viridis*^73^, ggplot2^74^, and *spThin*^75^. Georeferenced coordinate points of 117 *H. murphyi*, 43 *H. plumbeus*, and 22 *H. matannensis* were used for model building and projection. We used the 19 bioclimatic variables from WorldClim1^76^ at 2.5 min resolution as predictor variables and removed all layers that were correlated using a Pearson correlation with a threshold value of 0.80. We created a shape file of the complete geographic distribution of *Hypsiscopus* in QGIS v3.4.3 Madeira^77^ and used the R packages *rdgal* and *raster* to create a raster file with evenly spaced points at a resolution of 150 km to ensure coverage of all parts of the geographic range. The 19 bioclimatic variables with cell values of 0 were removed from the analysis. We retained 8 bioclimatic variables for ecological niche models: bio1 (annual mean temperature), bio2 (mean diurnal range (mean of monthly [maximum temperature—minimum temperature])), bio4 (temperature seasonality [standard deviation × 100]), bio10 (mean temperature of warmest quarter), bio12 (annual precipitation), bio15 (precipitation seasonality [coefficient of variation]), bio16 (precipitation of wettest quarter), and bio18 (precipitation of warmest quarter). We explored species-specific parameter tuning using five different feature class combinations: L, LQ, H, LQH, and LQHP (L = linear, Q = quadratic, H = hinge, P = product), with regularization multipliers of 1 through 5 for *H. murphyi*, *H. plumbeus*, and *H. matannensis* in ENMeval v2.0^62^. The best combination of feature classes was chosen based on the model with the lowest ΔAICc value (*H. murphyi*: LQHP, rm = 2; *H. plumbeus*: LQH, rm = 2; LQHP, rm = 2). Models of climate and habitat suitability were projected using the present uncorrelated bioclimatic variables. Eustatic sea level changes from glacial to interglacial conditions influenced the connectivity of suitable habitats by promoting the formation of interisland land bridges. To examine how past climatic changes may have affected predicted distributions of *Hypsiscopus*, we also generated niche model projections to the Last Glacial Maximum (LGM, ca. 22,000 years ago [kya]) conditions.

Additionally, we generated climate envelopes^78^, the range of conditions where *Hypsiscopus* are found throughout their range. Climate envelopes were constructed using the uncorrelated bioclimatic variables in our niche models: annual mean temperature, mean diurnal range, temperature seasonality, mean temperature of warmest quarter, annual precipitation, precipitation seasonality, precipitation of wettest quarter, and precipitation of warmest quarter. Using this framework, we were able to better understand the variation in environmental niche space occupied by *H. matannensis*, *H. murphyi*, and *H. plumbeus* across their range.

Finally, we used a COUE scheme (Centroid shift, Overlap, Unfilling, and Expansion)^79^ in the R package *ecospat* v3.2.2^63^ to quantify differences in occupied niches across species. This ordinatio-based approach can overcome biases associated with quantifying niche dynamics in geographic space, with differential sampling efforts and/or spatial resolution^80,81^. The analysis was performed using the entire spatial dataset of *H. murphyi* and *H. plumbeus*. We used the uncorrelated bioclimatic variables in our niche analyses (bio1, bio2, bio4, bio10, bio12, bio15, bio16, and bio18). This analysis requires that one species is designated as the ‘native’ species range, and the other as the ‘invaded’ species range. *Hypsiscopus* is hypothesized to have originated in Indochina^11^, and the distribution of *H. murphyi* is strictly in Indochina, while *H. plumbeus* is found in Indochina, Greater Sunda Islands, and Wallacea. Thus, we designated *H. murphyi* as the ‘native’ and *H. plumbeus* as the ‘invaded’ range. In this pipeline, we also quantified niche overlap using Schoener’s D^82,83^. Niche equivalency tests were performed to determine if the ecological niches of *H. murphyi* and *H. plumbeus* were significantly different or interchangeable from each other. To do this, pairwise comparisons of Schoener’s D overlap values were compared to a null distribution using 1000 random replicates^63^. Equivalency was determined if the observed Schoener’s D overlap value was significantly lower than the overlap values in the null distribution. Niche equivalency only assesses the niche space of the exact localities in our dataset, and not the surrounding environmental space. Thus, we also performed 1000 replicates of niche similarity tests to determine if environmental niche space is more similar than expected by chance. For niche equivalency and similarity, the null hypothesis is that the niches in the analysis are more different than expected from two random niches. Code and data for the ecological niche models and niche quantification is available in Supplementary Data D19–D26.

## Results

### Phylogenetic analyses

**Figure 2:**
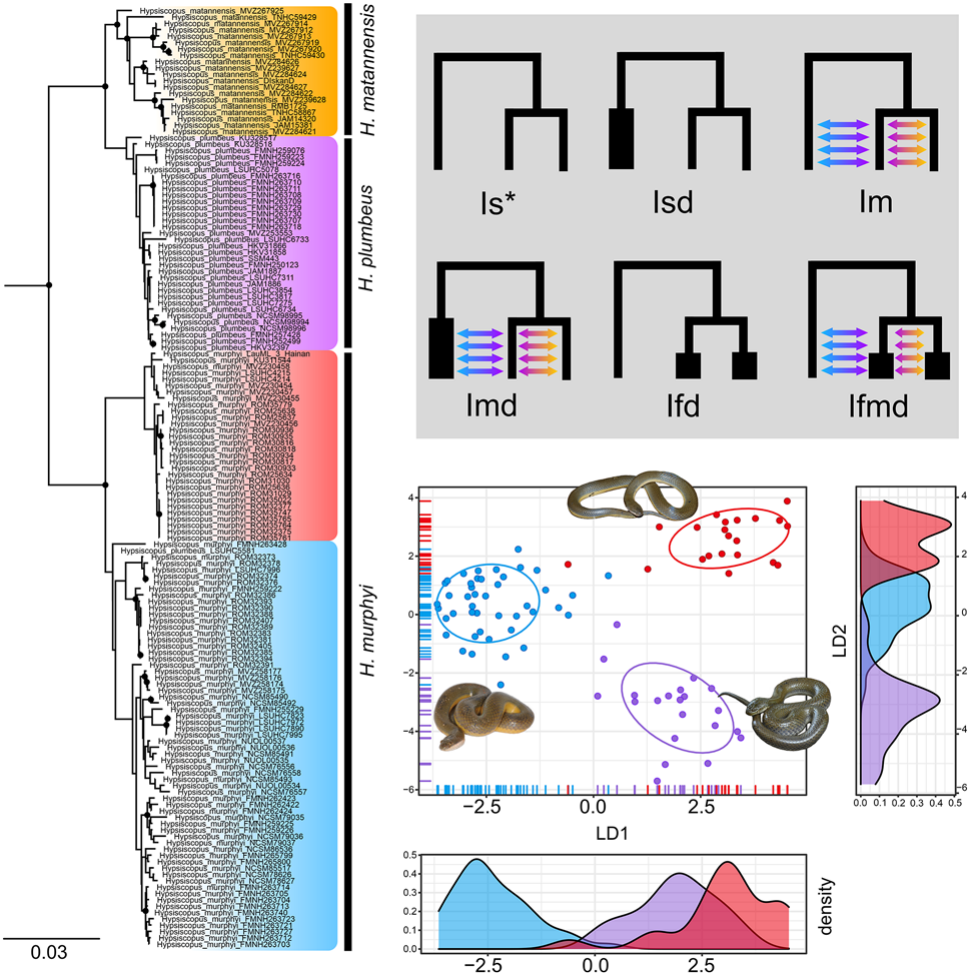
Molecular and morphological diversity in *Hypsiscopus*. Left) Phased concatenated phylogeny of *Hypsiscopus* (only one allele shown). *Hypsiscopus murphyi* is split into two subclades: East of the Red River (red) and west of the Red River (blue). Black circles at nodes represent strongly supported relationships (UFB ≥ 95 and SH-aLRT values ≥ 80). Scale bar in substitutions per site. Upper right) The six demographic scenarios tested in *PipeMaster*. Topology is based on the concatenated phylogeny (left), with both populations of *H. murphyi* collapsed into a single tip. The asterisk (*) shows the model with the highest support (Is). Bottom right) Linear discriminant analysis (LDA; 27 variables), showing three species of rice paddy snakes in morphospace. Points are shown with 68% Gaussian data ellipses. Density rugs (colored lines on axes) correspond to each point in morphospace. Density plots reflect the density of points in the plot for each species (colors correspond to the phylogeny).

All gene trees recovered *Hypsiscopus* as monophyletic, but showed varying levels of support and topological resolution (Supplementary Data D27–42). Our results are congruent with previously published phylogenies and species trees using multilocus and genomic data^7,11,27^. Cyt-b shows greater resolution than ATPase, yielding a topology in which *H. murphyi* is sister to the reciprocally monophyletic *H. matannensis* and *H. plumbeus* with strong support (Supplementary Data D26). The only species node in the cyt-b tree that is strongly supported is *H. plumbeus*. While ATPase inferred *H. murphyi* and *H. plumbeus* as monophyletic with strong support, *H. matannensis* was polyphyletic and interspecific support was weak. The nuclear genes PRLR, RAG2, AGBL5, and WFIKKN inferred trees with poor topological resolution and relationship support for both unphased and phased alignments. The only exception to this was WFIKKN, in which we recovered the expected relationships only recovered from the phased WFIKKN alignment. Both alleles of each sample were recovered close together in most trees. Though, in both the phased and unphased trees for RAG2 and WFIKKN2, a few samples of each taxon were recovered in a clade that is different than their conspecifics. The unphased and phased BNC1 trees recovered monophyletic groupings for all species, but only the split between *H. matannensis* and *H. plumbeus* was strongly supported. The phased VPS13B tree had stronger support at interspecific and species-level nodes compared to the unphased tree, and had one sample of *H. murphyi* and *H. plumbeus* found within *H. matannensis*. The concatenated trees recovered a strongly supported *Hypsiscopus* with *H. murphyi* sister to *H. plumbeus* and *H. matannensis*, but with low support (Fig. 2). The phased concatenated tree had better resolution than the unphased tree, which had samples that could not be placed in clades. One sample of *H. plumbeus* was recovered within the *H. murphyi* group (LSUHC 5581). This sample is represented by only PRLR, has a shorter sequence, and is less informative than other genes. Our phased concatenated tree was the most resolved tree, and thus we focus on this topology for our discussion. All trees can be found in Supplementary Data D26–D41.

The phased concatenated tree showed substructure in the populations of *H. murphyi* with the exception of one individual (Fig. 2; Supplementary Data D41). Samples from Hainan Island and the adjacent coast of Guangxi were sister to the individuals found at or near the Red River in Vietnam. The rest of the *H. murphyi* samples from west of the Red River group together, with many subclades that reflect individuals collected from the same or adjacent localities. We find that most of these smaller groups do not form a clade with other individuals that are geographically more distant in the same country, which is expected given the heterogeneous landscapes that traverse across political borders in Indochina. *Hypsiscopus murphyi* from Borneo were embedded within a clade consisting of individuals from Peninsular Malaysia, Cambodia, and Thailand. The newly sequenced samples of *H. matannensis* recovered geographically-based substructure for some samples (i.e., the Southwest Peninsula), but two subclades consisted of individuals from throughout Sulawesi. One sample of *H. plumbeus* from Sulawesi was recovered sister to samples from Peninsular Malaysia, Borneo, Cambodia, and Thailand.

### Morphological quantitative statistics

After removing non-normal and invariable characters, our PCA and LDA was performed on a dataset consisting of 27 variables: ventral scale count, color pattern transition, ventral color pattern, ventral tail color, number of left subcaudals, number of right subcaudals, head length, head width, left anterior chin shield length, right anterior chin shield length, left anterior chin shield width, right anterior chin shield width, left posterior chin shield length, right posterior chin shield length, left posterior chin shield width, right posterior chin shield width, left intergenial scale length, left intergenial scale width, right intergenial scale length, right intergenial scale width, infralabials contacting the posterior chin shields, total length, snout-vent-length, tail length, body width average at mid-body, and circumference at mid-body. The PCA recovered 42.4% and 11.3% of the variation explained by principal component 1 (PC1) and PC2 respectively. The PCA shows *H. murphyi* west of the Red River and *H. plumbeus* as distinct, with individuals of *H. murphyi* at and east of the Red River occupying more of the morphospace of *H. plumbeus* than the other *H. murphyi* group (Supplementary Fig. S3). The LDA had a classification accuracy of 97.7% (95% CI 91.85–99.72%; *p* =  < *2.2* × *10*^*–16*^). Examination of the confusion matrix revealed only two misclassified individuals, with one specimen of *H. plumbeus* being misclassified as *H. murphyi* from west of the Red River, and another specimen of the latter being misclassified as *H. plumbeus*. The LDA resulted in groupings congruent with the PCA. Along linear discriminant 1 (LD1, 58.6% percent group separation), the specimens of *H. murphyi* at and east of the Red River share a similar region of morphospace with *H. plumbeus*, while the *H. murphyi* population west of the Red River occupy a distinct morphspace (Fig. 2). Along LD2 (41.4% percent group separation), *H. plumbeus*, and both populations of *H. murphyi*, each occupy their own regions of morphospace (Fig. 2).

### Coalescent models and divergence dating

Bayesian Skyline Plots from BEAST2 revealed decreases in *N*_*e*_ ~ 250 kya in *H. plumbeus*, an increase in *N*_*e*_ at ~ 250 kya in *H. matannensis*, and an increase and then a decrease in *N*_*e*_ at ~ 2 mya and ~ 250 kya, respectively, for *H. murphyi* (Supplementary Fig. S1). Despite these observations in the BSP, the best supported demographic model in *PipeMaster* was the Is model (proportion of accepted simulations: Is = 0.5690; Im = 0.4237, Imd = 0.0073, Isd = 0, Idf = 0, Ifmd = 0; overall accuracy = 0.885). This suggests isolation with no migration or demographic change. The divergence time of the genus estimated from this model had a median of ~ 2.44 million years ago (mya) and a mean of 2.43 mya. The 95% confidence interval was between 1.4 and 3.48 mya. The divergence between *H. matannensis* and *H. plumbeus* had a median and mean of ~ 812 kya and ~ 821 kya, with a 95% confidence interval of 0–1.84 mya.

### Ecological niche modeling and niche quantification

**Figure 3:**
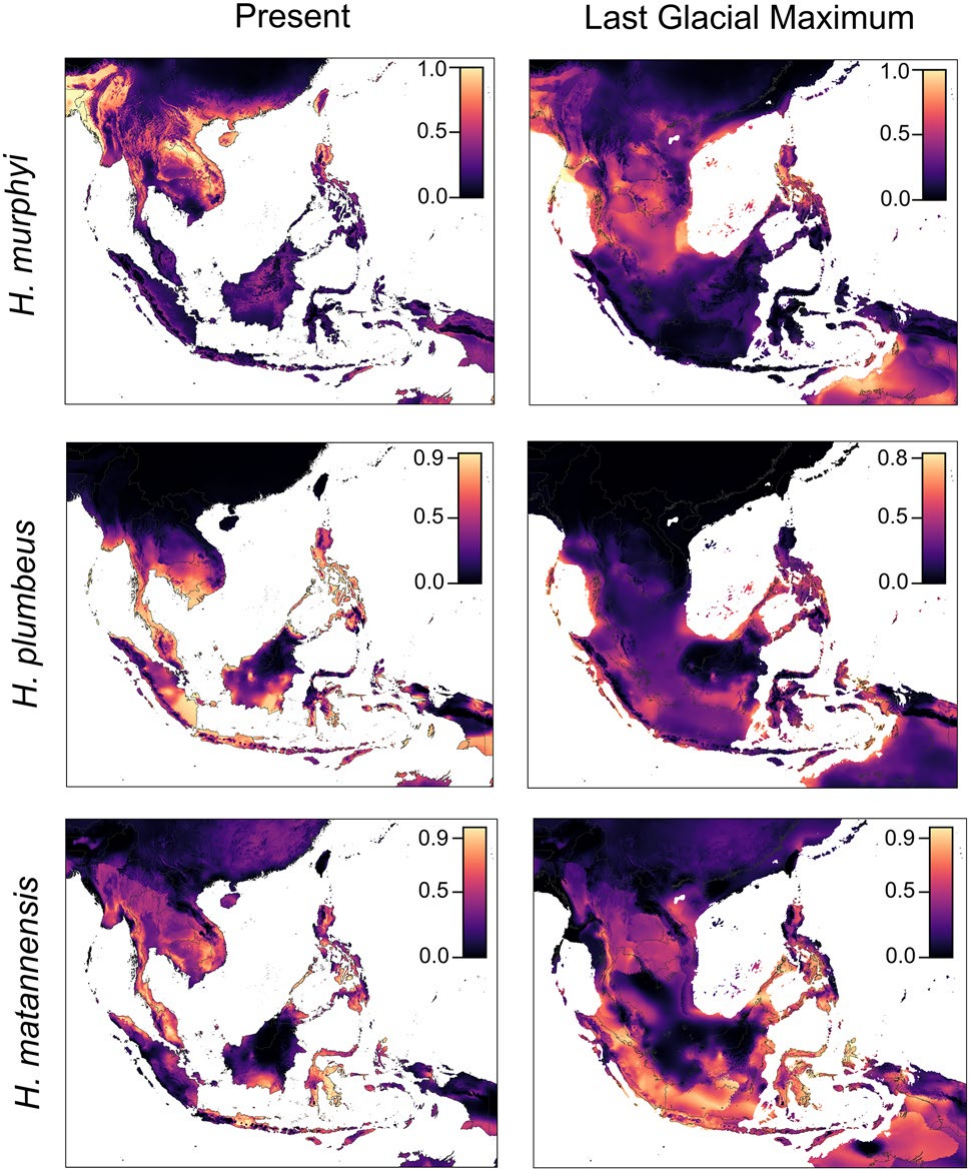
Ecological niche models of *H. murphyi*, *H. plumbea*, and *H. matannensis*. Colors represent habitat suitability, with warmer colors indicating higher habitat suitability based on environmental variables. Full-size images of each niche model can be found in Supplementary Figs. S5–S10.

Niche models for present-day projections are broadly reflective of the known distributions of each species. The present-day niche model for *Hypsiscopus murphyi*, only known from the Khorat Plateau and regions to its north, shows higher environmental suitability north of the Isthmus of Kra of mainland Southeast Asia, as well as in the eastern islands of the Lesser Sundas (and Timor Island) and the northern Philippines. This niche model shows the highest suitability in moderate to high elevation areas, including on the Khorat Plateau rim sites (Fig. 3). Contrarily, *H.* *plumbeus* indicates high habitat suitability near and south of the Khorat Plateau, including the Greater and Lesser Sunda Islands and the Philippines, with very low habitat suitability in moderate to high elevation areas such as the mountains of Sumatra, Java, the Lesser Sundas, Borneo, and Sulawesi, and low suitability on the Khorat Plateau, especially on rim sites. *Hypsiscopus matannensis* has the highest habitat suitability in Sulawesi and the Moluccas in the present-day model, as well as suitable habitat in the areas that *H. plumbeus* is present*.* However, *H. matannensis* showed higher habitat suitability in some high-elevation areas, such as the mountains of Sumatra, where *H. plumbeus* had a low likelihood of available niche space.

Our models projected to the LGM show that *H. murphyi* had the highest suitability at lower elevations in Indochina and the land bridge that formed between mainland Southeast Asia and the Greater Sunda Islands (Fig. 3). High suitability was also seen in the northern and central Philippines, and parts of Myanmar and South Asia. Lowest suitability is found in the region of Borneo, Java, and Sumatra, with the lowest likelihood found at high elevations in mountainous areas. Compared to its congeners, *H. plumbeus* showed a lower likelihood of suitability throughout the projected range. Moderate suitability was seen in low elevation areas, particularly where land is now above water, with the lowest values occurring in mountains regions. The LGM projections for *H. matannensis* showed a high likelihood of habitat suitability in the regions of Sumatra, Java, Sulawesi, southern Philippines, and Moluccas. Some parts of Borneo and other non-mountainous areas show moderate levels of habitat suitability. We note that the present-day models for *H. murphyi* and *H. matannensis* show high or moderate habitat suitability on the Khorat Plateau rim sites (but low suitability during the LGM), whereas *H. plumbeus* shows low suitability on the rim sites in both present-day and LGM models (Fig. 3).

The Area Under the ROC Curve (AUC) and Boyce Indices (BI) for niche models were (AUC/BI): 0.856/0.859 for *H. murphyi*, 0.825/0.513 for *H. plumbeus*, and 0.897/0.957 for *H. matannensis*. Amongst the uncorrelated variables, we found the greatest contributions (> 50%) for our models to be bioclim layers 4 (BIO4 = Temperature Seasonality [standard deviation × 100]), 1 (Annual Mean Temperature), and 15 (precipitation seasonality) for *H. matannensis*, *H. murphyi*, and *H. plumbeus*, respectively (Supplementary Fig. S4).

**Figure 4:**
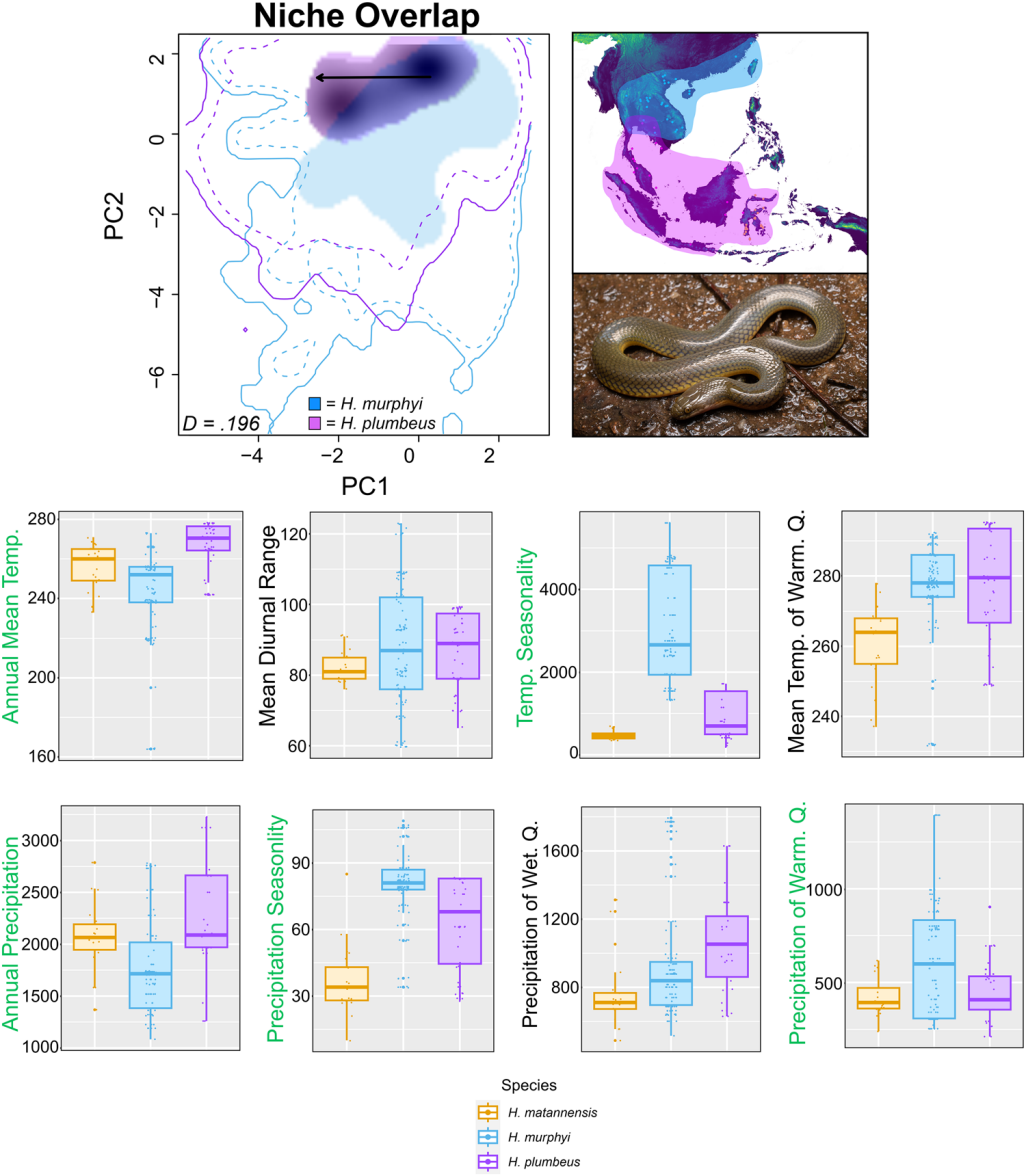
Niche quantification analysis between *H. murphyi* (light blue) and *H. plumbeus* (purple). Inset map shows total distribution of each species in Southeast Asia. Solid lines show the full background environmental space (100%) available for each species. Dashed contours represent 50% at each spatial scale. The light shaded areas illustrate the occupied niche space of *H. murphyi* and *H. plumbeus*. The dark shaded region highlights the overlap in niche space between the two species. Schoener’s *D* statistic is shown in the bottom left corner, indicating ~ 20% overlap in niche space. The black arrow shows a centroid shift of the ‘native’ niche (*H. murphyi*) to the ‘invaded’ niche (*H. plumbeus*). Photograph of *H. plumbeus* by Kenneth Chin. Climate envelopes of uncorrelated bioclimatic variables for all species of *Hypsiscopus* are shown as box plots, with green labels indicating a variable contribution > 10% in at least one of the niche models.

Evaluation of niche quantification in *ecospat* reveals a large overlap in the background environmental space available that *H. murphyi* and *H. plumbeus* can occupy (Fig. 4). *Hypsiscopus plumbeus* showed a more continuous occupied niche space when compared to *H. murphyi*, which occupied a disjunct niche space. Minimal overlap was observed between both species’ niches (Schoener’s *D* = 0.196). Our niche equivalency and similarity tests suggest that the niches of *H. murphyi* and *H. plumbeus* differed more than expected from two random niches (equivalency: *p* = 1; similarity: *p* = 0.43). We observed that there was a niche shift as *Hypsiscopus* diversified and expanded its range from Indochina into Sundaland and Wallacea (i.e., the distribution of *H. murphyi* to the distribution of *H. plumbeus*; Fig. 4).

## Discussion

We used genetic, morphological, and environmental data to investigate diversity in rice paddy snakes (Homalopsidae: *Hypsiscopus*) and test if their diversification coincided with the Khorat Plateau uplift in Thailand. Previous studies on homalopsid snakes have found similar phylogenetic and morphological patterns^11,27^. In this study, we increased genetic and geographic sampling density, and examined alternative population histories under a coalescent simulation framework. Multiple data types identified previously undocumented patterns of interspecific and intraspecific diversity in this genus. Our results suggest both geological processes and environmental factors have led to the present-day patterns of species diversity and their respective distributions. We provide evidence that the Khorat Plateau was likely a driver of diversification in rice paddy snakes. Additionally, we show that environmental differences may have maintained differentiation as lineages diverged from one another, and niche shifts have taken place during speciation.

### Molecular and morphological diversity of Hypsiscopus

The concatenated phylogeny constructed with phased genetic data recovered each species as monophyletic. This topology is consistent with evolutionary relationships estimated previously, using both multilocus and genomic datasets^11,27^. Our study includes newly added samples of *H. matannensis* from Sulawesi and *H. murphyi* near and east of the Red River in northern Vietnam (called the Red River population hereafter). The Red River population is monophyletic and sister to all other *H. murphyi* (Fig. 2). The Red River is considered to be a major biogeographic barrier^39^, with known divergences occurring here for birds^84^, frogs^39,85–87^, and lizards^88^. Within this Red River population, we observe substructure between samples from Hainan + Guangxi and those in Vietnam. One sample from the Vietnam clade is found on Hainan, which is not surprising given the land bridge between mainland China and Hainan Island during sea level minima in the Pleistocene and the close proximity of the Leizhou Peninsula to Hainan (~ 20 km).

Although the new samples of *H. matannensis* revealed substructure in the concatenated phylogeny (Fig. 2), the clades recovered did not reflect what may be expected based on Sulawesi’s geological history ^89,90^. Sulawesi’s arms (peninsulas) that branch from the island’s center (the Central Core) contain multiple potential contact points of paleo-islands when Sulawesi was partially submerged over the last 20 million years. Many of the inter-arm bays shrank in the last 4 million years leading to the coalescence of paleo-islands of Sulawesi, especially during the Pleistocene^90–93^. Many vertebrates show geographic-based population structure that reflects this history, such as *Draco* lizards^94^, *Bunomys* rodents^95^, *Limnonectes* frogs^96^, and studies on macaque monkeys and Celebes Toads (*Ingerophrynus celebensis*) have led to the establishment of areas of endemism (AOEs^89,97–99^), which are not reflected in Sulawesi rice paddy snakes. Some of our samples of *H. matannensis* from multiple peninsulas or islands showed minimal, intraspecific divergence and are recovered in multiple clades (Supplementary Fig. S11). Although further work and sampling is needed to thoroughly assess the biogeography of rice paddy snakes on Sulawesi, studies on lizards^100^, shrews^101^, and snails^102^ have also found distributions that are partially incongruent with the monkey and toad AOEs or known historical geographic units (e.g., paleo-islands). This might be expected for more recent colonizations of Sulawesi that happened after the merger of paleo-islands had already taken place. Interestingly, the last paleo-island to merge was the southwest peninsula, which is the only place we detect any phylogenetic structure.

*Hypsiscopus* are absent from Australia, New Guinea, the Lesser Sunda Islands, and the Philippines. Thus, colonization of Sulawesi from the north, south, or east is unlikely. The Makassar Strait that separates Sulawesi and its west-neighboring island Borneo forms a strong barrier for reptiles and amphibians, with ~ 100 genera from Borneo being absent from Sulawesi^94^. Vicariance between Sulawesi and Borneo is ruled out, as this deep straight opened ~ 45 mya^103^, significantly older than the crown date of *Hypsiscopus*. Thus, an over-water dispersal might be the most likely dispersal scenario into Sulawesi. Many species, such as terrestrial *Sphenomorphus* skinks^104^, *Draco* lizards^105^, *Cyrtodactylus* geckos^106^, and frogs in the genus *Limonectes*^96^ have been suggested to have dispersed between islands on the Lesser Sunda Arc (Lombok, Sumbawa, Komodo, Flores, and Lembata). However, although our phased concatenated tree supports a Southwest colonization of Sulawesi (likely from Selayar Island, for which samples here cluster with those of the Southwest Peninsula), a southern dispersal from the Lesser Sunda Islands is considered improbable pending the discovery of *Hypsiscopus* from these islands. Future studies that include samples from the Central Core of Sulawesi and more sampling of the Greater Sunda Islands will likely identify colonization routes into Sulawesi.

Phenotypic similarity is expected to be greater within species than between species. However, underlying species divergence is sometimes masked by conserved or parallel phenotypes in different taxa with wide distributions^23,107,108^. The morphological differentiation of *H. plumbeus* and both populations of *H. murphyi* is quantitatively supported by the PCA and LDA (Fig. 2; Supplementary Fig. S3), with nearly perfect group classification accuracy in the LDA (see *Results: Morphological Quantitative Statistics*). The Red River population of *H. murphyi* is phenotypically distinct from its conspecifics, having a gradual color change from dorsal to ventral scales and half-moon patterns on each ventral scale. This Red River population of *H. murphyi* is more phenotypically similar to *H. plumbeus* than to other *H. murphyi*. It is unclear if the conserved color pattern in Red River *H. murphyi* and *H. plumbeus* is due to incomplete lineage sorting of alleles related to color pattern or due to convergence in the environmental niches they occupy, as these species exist in both mainland (Indochina and Malay Peninsula) and island (Hainan, Taiwan, Greater Sundas) localities. Research on microhylid frogs also found in this region has shown ecological differences between the east and west sides of the Red River^39^, though, our niche models show a difference in elevation suitability between *H. murphyi* (both populations) and *H. plumbeus*, and the Red River population of *H. murphyi* is at an elevation more similar to the distribution of *H. plumbeus* (see below). Both niche conservatism and niche divergence could drive the observable patterns seen in species. Niche conservatism can drive phenotypic conservatism, and both niche conservatism^109,110^ and niche divergence^109,111,112^.

### Geological and environmental drivers of diversity

Our study expands the sampling density of prior studies and allowed us to test biogeographic hypotheses regarding *Hypsiscopus* diversification. While Bernstein et al.^11^ used ~ 4800 nuclear loci in their study, only 14 individuals across all three species were included. Our demographic models using 149 individuals support the hypothesis that *Hypsiscopus* diversified ~ 2.5 mya, suggesting the Khorat Plateau influenced the diversification of rice paddy snakes in Southeast Asia. Demographic models showed no evidence of migration or change in *N*_*e*_; this, and the distribution of outgroups, suggests that *Hypsiscopus* diversified from mainland Southeast Asia, eastward through the Greater Sunda Islands, eventually colonizing Sulawesi.

Pleistocene sea level fluctuations are one of the prevailing paradigms of diversification of land vertebrates—via sea level vicariance—in Southeast Asia^113,114^. Phylogeographic patterns that temporally coincide with sea-level minima and maxima have been observed in spiders^115^, freshwater fish^116,117^, birds^118^, frogs^119,120^, gekkonid lizards^121^, and shrews^122^. However, inland geological influences are a more likely explanation for some taxa. These include paleo-rivers and drainage basins^39,88,123,124^, mountains^125^, and Quaternary landscape dynamics^126^. Tectonic uplift events, such as those that formed the Khorat Plateau, can also affect the distributions of elevation-sensitive species.

Although our BSP suggests changes in *N*_*e*_ may have occurred in the Pleistocene for each species (Supplementary Fig. S1), our demographic model results suggest that the much older Khorat Plateau uplift event might have initiated the divergence of rice paddy snakes in Southeast Asia and that no demographic changes have taken place (or these signatures were not strong enough to be detected). We acknowledge that our BSP were estimated using only mitochondrial data, but the demographic models take information from all loci (nuclear and mitochondrial) which show a better fit for a model that does not allow gene flow. Additionally, the faster rate of mitochondrial genes better captures potential demographic change in the BSP in the recent time frame of the biogeographic story we are investigating. Thus, we favor the results of the demographic models that support no change in *N*_*e*_ over time. The Khorat Plateau is not particularly high in elevation (and geologically, is actually a basin with a depression in the middle, but we refer to it as a plateau due to its formal name in the literature). It consists of the Lower Mekong River, Phanom Dong Rak Mountains, and Phetchabun Mountains on the eastern, southern, and western sides, respectively, which reach elevations of 200–1100 m above sea level. Although the Khorat Plateau rim sites are not high in elevation, homalopsids are generally considered a low-elevation group (Murphy, 2007). Additionally, studies on the Khorat Plateau show differing densities of some mud snake species (*Enhydris* spp., *Subsessor bocourti*, *Erpeton tentaculatum*, *Homalopsis buccata*, *Hypsiscopus plumbeus* [sensu lato]) on the plateau in comparison to outside of the plateau, and no mud snakes of any species were found on the rim sites^127^. Furthermore, morphological differences in size and extent of sexual size dimorphism are significantly different when comparing populations on and off the plateau^127^. It is clear that the plateau leads to at least some inhibition of migration of mud snakes—a general result which has been seen in other vertebrates, including volant species like birds of the genus *Alophoixus*^128,129^, emydid turtles (*Malalemys*^130^), and potentially in Figs. ^131^. It is worth noting that the basin is tilted towards the south and the east with an average elevation of 200 m, and the rim sites act as physical barriers that weaken the effects of the monsoons that commonly impact this region. This makes the plateau the hottest and driest area in Thailand with the greatest seasonal flux between wet and dry seasons and contains low-fertility soils^40,132^. Thus, the Khorat Plateau not only represents a physical barrier, but may also serve as an ecological barrier between species as well. Environmental differences may affect the distributions of rice paddy snakes, and we see evidence of this in our niche modeling and quantification as well (Fig. 3).

Our ecological niche models reflect the currently known distributions of rice paddy snakes and suggest that their present-day distributions occupy different niches, most notably in elevation. *Hypsiscopus murphyi* showed higher suitability in moderate elevation areas, including the Khorat Plateau, with lower suitability at very high or very low elevations and south of the Isthmus of Kra biogeographic barrier on the Malay Peninsula. In contrast, niche models showed higher suitability at very low elevations for *H. plumbeus* and *H. matannensis* (except on Sulawesi for *H. matannensis*), with the lowest suitability for both species in the chain of volcanoes that span the Greater and Lesser Sunda Islands and the high mountains of central Borneo (~ 1000–4000 m elevation). This suggests environmental differences in elevation, as well as annual mean temperature, mean diurnal range, temperature seasonality, mean temperature of warmest quarter, annual precipitation, precipitation seasonality, precipitation of wettest quarter, and precipitation of warmest quarter (Supplementary Fig. S3) likely impact the current distribution of *Hypsiscopus.* The ENM projections to the LGM showed differences in habitat suitability between all species, suggesting that environmental differences may be responsible for historical diversification processes leading to the extant species richness of these snakes. Our niche quantification analysis supports the interpretation of niche differentiation between *H. murphyi* and *H. matannensis*, reinforcing the ecological differentiation observed between these closely related taxa, possibly via a gradual evolutionary niche shift in *Hysiscopus* as the ancestors of today’s lineages diversified from west to east (Fig. 4).

We acknowledge that demographic models are heavily reliant on input data and parameter values, and the resulting divergence dates will affect interpretation of biogeographic scenarios. Future studies on this group should focus on filling sampling gaps, particularly around the plateau. While we use the densest sampling possible, obtaining genomic data for more individuals of *H. plumbeus* and *H. murphyi* would provide a greater degree of confidence in divergence estimates and determining the most likely speciation scenario for *Hypsiscopus*. We do not perform strict gene flow analyses in this study given the limited sampling of both taxa in the overlapping areas of their distribution. However, we attempt to account for these limitations by using high numbers of samples and testing demographic models that allow or disallow gene flow. Unlike more exploratory frameworks for interpreting biogeographic scenarios^7,11^, we employ a hypothesis/model-testing framework to identify the most likely speciation scenario. Given the corroboration of morphological groupings with molecular data, the coincidence with species distributions and geological features in the region of the plateau, and supporting differentiation as shown by our niche analyses, we are confident in our interpretations based on our data in this study. Nonetheless, future studies that include higher locus and taxon sampling that can better assess gene flow and fine-scale population structure will be important for finding congruent (or alternative) divergence histories of *Hypsiscopus*.

Our work suggests that the diversification of *Hypsiscopus* may have been influenced by multiple processes, with an initial vicariant divergence due to the Khorat Plateau uplift, and then distinct environmental features (both near and far from the plateau) helped maintain their divergence. Environmental differences in mainland Southeast Asia have led to the diversification of microhylid frogs^39^ and birds^133,134^, many of the latter having near-identical distributions as *H. plumbeus*. This scenario contrasts greatly from the often-referenced Pleistocene sea-level fluctuations that have led to the diversification of many groups in Southeast Asia^111^. Our study identifies other modes of diversification in this model region, and suggests the need to revisit biogeographic hypotheses and determine if terrestrial vs. marine changes (or other factors) of historical landscapes have influenced extant diversity and population structure, as shown in recent works on homalopsids^11^. We highlight the importance of utilizing multilocus datasets and numerous methods and data types to test fine-scale biogeographic hypotheses and identify patterns of differentiation in poorly studied, yet widespread species. The evolutionary history of rice paddy snakes has only recently been studied^27^. Our sampling of nearly 200 individuals provides a basis for future studies to investigate broad and fine-scale processes such as gene flow and dispersal—and to characterize the colonization routes both may take throughout the complex geography of this environmentally heterogeneous region. Our empirical approach in this study allows us to ultimately understand dynamic geological and environmental processes that continue to shape Southeast Asia, as well as the biodiversity generated, partitioned, and maintained on either side of Wallace’s Line.

### Supplementary Information

Supplementary Information.

## Data Availability

New molecular data for cyt-b, ATPase, PRLR, VPS13B, WFIKKN, AGBL5, BNC1, and RAG2 generated and or analyzed during the current study are available on NCBI GenBank under the Accession numbers OM479857–OM480509. Morphological data and all GenBank accession numbers are available in Supplementary Table S1. All code and respective data files for this study are available at https://github.com/jbernst.
